# Current Review of Monoclonal Antibody Therapeutics in Small Animal Medicine

**DOI:** 10.3390/ani15040472

**Published:** 2025-02-07

**Authors:** Jianzhong Wang, Xueying Zhou, Sara T. Elazab, Jian Huang, Walter H. Hsu

**Affiliations:** 1 Shanxi Key Laboratory for Modernization of TCVM, College of Veterinary Medicine, Shanxi Agricultural University, Taigu 030801, China; jianzhongwang@cau.edu.cn; 2 Department of Veterinary Clinical Science, College of Veterinary Medicine, China Agricultural University, Beijing 100107, China; xueying@cau.edu.cn; 3 Department of Pharmacology, Faculty of Veterinary Medicine, Mansoura University, Mansoura 35516, Egypt; sara.taha@ymail.com; 4 Institute of Qinhai-Tibetan Plateau, College of Animal Husbandry and Veterinary Medicine, Southwest Minzu University, Chengdu 610041, China; jhvet03@sina.cn; 5 Department of Biomedical Sciences, College of Veterinary Medicine, Iowa State University, Ames, IA 50011, USA

**Keywords:** monoclonal antibody, mAb therapy, immunotherapy, chronic disorders, nerve growth factor, canine interleukin 31, lymphoma, osteoarthritis, atopic dermatitis, pet animals

## Abstract

Monoclonal antibody therapy has been a cornerstone of human healthcare for nearly four decades, effectively treating a wide range of diseases including cancers, autoimmune disorders, and inflammatory conditions. However, its application in veterinary medicine is a relatively recent development, offering a promising therapeutic approach for managing chronic diseases in small animals. Dogs and cats, like humans, suffer from chronic conditions such as cancer, arthritis, allergies, and chronic pain, which mAb therapy could potentially address. This review aims to explore the therapeutic potential of mAb therapy in small animal medicine, focusing on currently authorized products, including their mechanisms of action, clinical efficacy, and safety concerns. A comprehensive review of the literature was conducted to evaluate the use of mAbs in veterinary medicine, specifically in the treatment of chronic disorders. While mAb therapy has shown significant benefits in human healthcare, challenges remain in its application to veterinary practice, including safety concerns and the limited availability of approved products. Despite these challenges, mAb therapy holds great promise for improving the management of chronic diseases in animals, with future research and development potentially expanding its clinical use.

## Simple Summary

Monoclonal antibodies (mAbs) have been a cornerstone of human healthcare for nearly four decades, with applications in oncology, autoimmune diseases, and inflammatory conditions. However, the introduction of mAb therapy in veterinary medicine, especially for small animals, represents a relatively recent but promising therapeutic approach. As dogs and cats commonly suffer from chronic disorders such as cancer, arthritis, allergies, and chronic pain, mAb therapy has the potential to offer these animal patients the same benefits as those observed in human treatments. Despite the significant success of mAbs in human medicine, their use in veterinary medicine remains limited, with only a handful of products approved and commercially available. In this review, we explore the therapeutic potential of mAbs in veterinary medicine, detailing currently authorized products, including bedinvetmab (Librela™, Zoetis), frunevetmab (Solensia^®^, Zoetis), and lokivetmab (Cytopoint^®^, Zoetis). We also address the safety concerns and unique challenges associated with mAb therapy in veterinary contexts, providing insight into the future development and application of these therapeutics in small animal healthcare.

## 1. Introduction

Monoclonal antibodies (mAbs) are an important class of medications that have been proven invaluable for the therapeutic strategies for cancers, autoimmune diseases, and other indications (such as infectious diseases, inflammatory disorders, and allergies) [1]. MAb therapy has been an established therapeutic strategy in human healthcare for nearly four decades, offering a diverse spectrum of treatment options for various diseases. Hundreds of mAbs are currently in clinical development for diseases and conditions ranging from inflammation to cancers [2]. Since 2015, 23% of drugs approved by the FDA have been antibodies, and on average, four mAbs enter the human drug market per year [2]. As of 31 December 2023, the FDA had approved 163 therapeutic mAbs for human use (FDA. “Approved Drugs with Therapeutic Monoclonal Antibodies”. Available at: https://www.fda.gov/drugs. Accessed on 27 January 2025). Therapeutic antibodies are highly successful biopharmaceuticals and were fourth among the ten top therapeutics by sales in 2021 [3]. MAbs made up the largest share of therapeutic antibodies in biopharmaceuticals market sales in 2023, at 35% [4].

**Figure 1:**
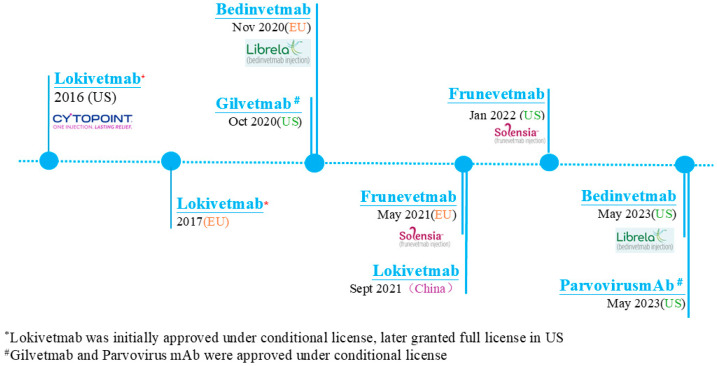
A timeline of mAbs in veterinary medicine.

Veterinary mAbs represent a novel therapeutic class, arriving on the scene about three decades later than their human counterparts [4]. To date, only five mAbs are in the animal drug market (Figure 1 and Table 1): bedinvetmab (Librela^®^, Zoetis), frunevetmab (Solensia^®^, Zoetis), lokivetmab (Cytopoint^®^, Zoetis), gilvetmab (Merck Animal Health), and canine parvovirus monoclonal antibody (CPMA, Elanco); the latter two have received conditional approval MAbs are now being used for chronic conditions in dogs and cats. In this review article, we review the use of mAbs as biological remedies in the veterinary field with an emphasis on the authorized and commercially available products.

## 2. How Are MAbs Made?

MAbs, initially developed in mice in 1975, are produced either from single B-lymphocyte clones in mice or through recombinant engineering [5]. These antibodies are monovalent and specifically bind to target molecules such as cytokines, receptors, or cells, blocking their activity upon binding [5]. This contrasts with polyclonal antibodies (pAbs) which originate from a diverse mixture, capable of recognizing and binding to multiple epitopes of a single antigen [5]. Since Köhler and Milstein introduced the hybridoma technique in 1975 [6], advancements in mAb generation technologies have expanded tremendously. These now include techniques such as utilization of transgenic animals to produce fully humanized, caninized, and felinized mAbs, ribosome display methodology [7], DNA recombinant antibody techniques [8], antibody phage display [9,10,11], and isolation of single B-lymphocytes for subsequent clonal production [12,13].

**Figure 2:**
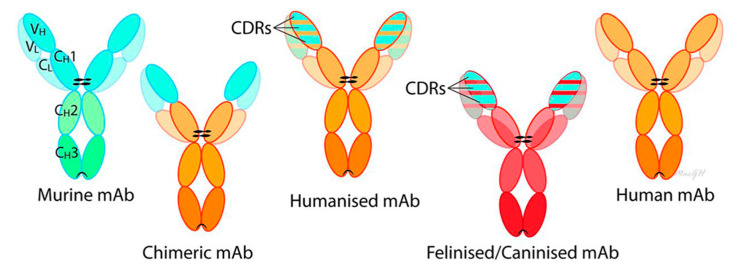
Composition of antibodies. Antibodies are large glycoproteins made of two heavy and two light chains, each with variable (VH, VL) and constant (CH, CL) domains (adapted from Masataka et al. [20]). The variable domain contains six hyper-variable loops (CDRs) that determine antigen specificity, while the constant domain is the same within an antibody isotype (e.g., IgG, IgM) but differs across isotypes. The Fc region of the constant domain activates immune responses. Murine mAbs come from cloned mouse B-lymphocytes, with most therapeutic mAbs being IgG isotypes. Chimeric mAbs combine murine variable regions with human constant regions, while humanized mAbs retain only the murine CDRs. Fully human, caninized, and felinized mAbs contain no murine sequences, and species-specific antibodies can be developed through processes like PETization, which aligns them with species-specific immunoglobulin libraries.

Antibodies derived from different species (xenogeneic antibodies) tend to provoke a stronger immune response compared with those derived from the same species (syngeneic antibodies) [14], and therefore, antibody engineering techniques have been created to reduce the likelihood of immune responses (immunogenicity) [15]. To adapt these antibodies for use in humans, a process called “humanizing” or “humanization” has been employed. By substituting mouse-derived amino acids with human sequences, the immunogenicity of mAbs has been significantly reduced. Similarly, when a rodent mAb is modified for treatment in cats/dogs, it is termed “felinized”/“caninized” to indicate its compatibility with the feline/canine immune system (Figure 1). Over the past 25 years, various approaches such as chimeric, humanized, or felinized/caninized, and fully humanized or felinized/caninized mAbs have been produced to decrease immunogenicity (Figure 1) [16]. Chimeric mAbs, comprising approximately three/four human (or canine/feline, as required) sequences, are generated by fusing murine-derived variable regions with human/canine/feline antibody constant regions [17]. Subsequently, the “humanization/felinization”/“canonization” process retains only the complementarity-determining regions (CDRs), resulting in constructs that are approximately 95% human (or canine/feline, as required) [18]. Fully humanized (or caninized/felinized) mAbs, devoid of murine sequences, have been produced using transgenic mice and phage technologies. Despite the efforts to minimize immunogenicity, factors such as route of administration, treatment paradigm, and concurrent immunosuppressive therapy influence the immune response in clinical practice. Undesirable immune responses can neutralize the therapeutic effects of mAbs and adverse reactions, emphasizing the importance of considering immunogenicity in their therapeutic use. Additionally, some mAb immunogenicity appears to be idiotypic, where patients may exhibit differential reactivity to different chimeric mAbs [19]. The antibody production technologies discussed are depicted in Figure 2 [20].

Figure 2 illustrates techniques for generating antibodies of various speciation levels and includes the following methods: (1) Antibodies can originate from mice or other laboratory animals that have been immunized with the target antigen. If the mouse has not been genetically modified to express the immune system of another species (e.g., xenomouse), antibodies derived from laboratory animals will typically be ‘speciated’ (e.g., humanized). (2) Antibodies can originate directly from the species of interest. In this scenario, phage display technology can be utilized to select the desired antibody from the diverse pool available in any animal. Antibodies derived using this approach are termed ‘humanized’, ‘caninized’, etc. 

Several companies have either developed or are currently developing technologies to produce species-specific antibodies designed for veterinary use. For instance, Nexvet Biopharm created a technology to develop anti-NGF mAbs specifically tailored for dogs and cats. This method involves using complementary DNA libraries to compare variations in the heavy and light chain sequences of mAbs between donor species (such as humans or rodents) and the target species. By identifying the minimal number of changes needed at each position in the amino acid sequences, the donor mAb variable region sequences are converted to mAb sequences containing only amino acids found in the target species. Subsequently, necessary amino acid substitutions are made, replacing them with the most similar amino acids from the target species [20]. 

This process results in the generation of mAb sequences that are entirely species-specific, thereby minimizing the risk of immune rejection while maintaining high affinity and potent bioactivity. Nexvet successfully converted the rat anti-NGF mAb (αD11) into caninized and felinized anti-NGF mAbs, aimed at treating painful conditions, including osteoarthritis (OA), in dogs and cats (see Section 5.2) [20].

## 3. Pharmacokinetics (PKs) of MAbs

The PK features of mAbs are different from those of small molecule drugs [21]. Therapeutic mAbs belong to a class of antibody drugs characterized by their high uniformity and specific pharmacological effects targeting a single epitope achieved through molecular biology technics. Notably, mAbs have a much higher molecular weight and polarity compared with small molecule drugs, resulting in unique PK properties [21]. Typically, injected mAbs display biphasic PK profiles, i.e., a rapid distribution phase followed by a slow elimination phase. Other mAb-specific PK properties involve their restricted distribution in the vasculature and interstitial space due to their large size and polarity, prolonged half-lives (~11–30 days in humans) because of neonatal Fc receptor (FcRn)-mediated recycling, and nonlinear PKs due to target protein-mediated clearance [21]. The following discussion will focus on the mechanisms and characteristics of mAb PKs with respect to absorption, distribution, and elimination.

### 3.1. Route of mAb Administration and Absorption

To ensure their efficacy, therapeutic mAbs are administered via IV, IM, or SC routes. MAbs have extremely low oral bioavailability (typically <1–2%) [22]. This is attributed to several factors: (1) protein denaturation in the acidic gastric environment, (2) degradation by hydrolytic enzymes in the gut [23], and (3) limited absorption due to the large size and polarity of proteins [24]. As a result, most mAb drugs are administered parenterally. 

### 3.2. Distribution of MAbs

In the steady state, population estimates for the volumes of distribution in the central (Vc) and peripheral (Vp) compartments are generally small. In humans, the median values are approximately 3.1 L (range 2.4–5.5 L) and 2.8 L (range 1.3–6.8 L), respectively [25]. This limited distribution to the vascular and interstitial spaces is due to the size and polarity of mAbs, which restrict their ability to leave the vascular space [25,26].

Factors influencing mAb distribution include diffusion, tissue removal, and biophysical properties such as molecular charge and hydrophilicity [27]. In addition, the specific binding of an antibody to its antigen, as well as factors such as binding affinity, receptor expression, receptor turnover kinetics, and antigen–mAb interaction, can impact distribution [28]. For example, an mAb, following IV administration, is distributed from the vascular space to the interstitial space primarily through fluid flow and binding to cells, or via receptor-mediated endocytosis, phagocytosis, or pinocytosis [29].

### 3.3. Metabolism and Elimination of MAbs

Unlike conventional drugs, mAbs do not require biotransformation for inactivation or excretion [26]. In addition, due to their high molecular weight (~150 kDa) which is higher than the glomerular filtration threshold (~55 kDa), antibodies cannot be excreted by the kidneys in their intact form, nor are they metabolized by hepatic enzymes [26]. Instead, they follow pathways similar to natural physiological protein products [26]. Intracellular catabolism of mAbs occurs within lysosomes, where they are degraded into amino acids that can be recycled for protein synthesis or excreted via the kidneys. This inactivation process provides mAbs with a significant advantage over conventional drugs, as the likelihood of adverse drug interactions when co-administered with other drugs is low [26]. The principal elimination mechanisms for mAb drugs include antigen-mediated elimination, pinocytosis, Fc-gamma receptor (FcγR)-mediated elimination, and neutralization of anti-drug antibodies. Also, the specific binding between an mAb and its target protein can greatly reduce the elimination of the mAb drug [26].

The half-life of each specific antibody will define the dosing schedule and mode of delivery [20]; MAbs have an elimination half-life of 7–28 days (mostly 21 days) in humans [20]. However, the half-life of some mAbs (such as frunevetmab) in cats and dogs averaged 11 days (ranging 7–15 days) [30]. MAbs in circulation that bind to FcRn on endothelial cells are shielded from degradation by lysosomal enzymes, allowing them to be recycled back into the plasma. This recycling mechanism contributes to the extended half-life of mAbs [30].

## 4. Safety of MAb Therapy

Like any emerging therapeutic strategy, the use of biopharmaceutics raises concerns about the safety of these compounds. Practitioners often express apprehensions regarding a wide range of potential adverse reactions associated with the mAb therapy [31]. However, such concerns are largely unfounded, given the highly specific targeting of mAbs and their unique metabolic characteristics [31]. By virtue of their lack of intracellular activity, adverse reactions can be more precisely predicted prior to clinical investigations using the expected blockade of the target protein. It is well-known that mAbs, in general, offer a safer treatment option than conventional drugs. This favorably low risk–benefit ratio is supported by the reality that the probability of mAbs receiving regulatory approval is about four times higher than that of other newly developed pharmaceutics [31].

Typically, the overall safety of a particular mAb is largely determined by the type of treatment, the target of the therapy, and the level of speciation [32]. The speciation refers to the degree to which an antibody specifically recognizes and binds to its intended target molecule. Adverse reactions that have been observed with therapeutic mAbs in humans include injection site discomfort, lethargy, fever, and gastrointestinal disturbances [33]. The effects of mAb therapies are generally predictable based on the mechanism and target of each specific product. Some patients may experience reactions such as rashes, hemorrhage, or bruising, all of which are dependent on the mAb’s target. The long-term adverse effects of currently available mAbs will become clearer with future research, which will also likely lead to new and improved forms of these therapies. Compared with conventional pharmaceutics, mAb therapies pose fewer safety risks related to overdosing, drug interactions, underlying conditions, or contraindications such as species or age [32]. A crucial aspect of developing effective mAb therapies lies in comprehending the pathophysiology of the targeted disease. In addition, understanding the role of the specific cytokine or protein being targeted is essential in assessing the safety of mAb therapy. In cases where the targeted cytokines serve multiple physiological roles, there is a potential for adverse effects when specific cytokines are blocked [34]. For instance, IL-31, a cytokine mediating inflammatory responses, plays a protective role in multiple organ systems, including the lungs, gastrointestinal tract, and nervous system [35]. It helps protect these tissues [35]. However, when free IL-31 levels are drastically reduced due to therapeutic use of IL-31, it may lead to infections [36]. In the nervous system, diminished IL-31 could affect nerve signaling or sensory function [37]. Therefore, while targeting IL-31 may provide therapeutic benefits, careful consideration is needed to balance efficacy with the risk of disrupting its protective roles in essential physiological processes.

Therapeutic proteins, including mAbs, have the potential to induce an immune response in the host, resulting in the production of antibodies against the therapeutic protein itself. These host-derived antibodies, often referred to as anti-drug antibodies, can include a subset known as neutralizing antibodies (NAbs). NAbs bind to the therapeutic antibody, thereby potentially reducing its efficacy. Moreover, NAbs may accelerate the clearance of the therapeutic antibody from the body and, in severe cases, lead to anaphylaxis, including anaphylactic shock [38]. Understanding the underlying mechanisms that contribute to mAb immunogenicity is important for enhancing their therapeutic effectiveness and safety. It is essential to develop robust strategies to identify and mitigate the immunogenic risk associated with mAb therapies.

## 5. MAbs in Veterinary Medicine

MAbs have revolutionized veterinary medicine by offering targeted therapeutic options for various medical conditions in companion animals. New entrants with innovative treatment approaches are reshaping the market. These include as lokivetmab (Cytopoint^®^ by Zoetis, Parsipanny, NJ, USA), frunevetmab (NV-02, as Solensia^®^ by Zoetis), bedinvetmab (Librel^®^ by Zoetis), gilvetmab (Intervet Inc., Rahway, NJ, USA, Merck Animal Health), and CPMA (Elanco, Indianapolis, IN, USA) (Figure 1 and Table 1). 

### 5.1. MAbs Against Atopic Dermatitis (AD) in Dogs—Lokivetmab

**Figure 3:**
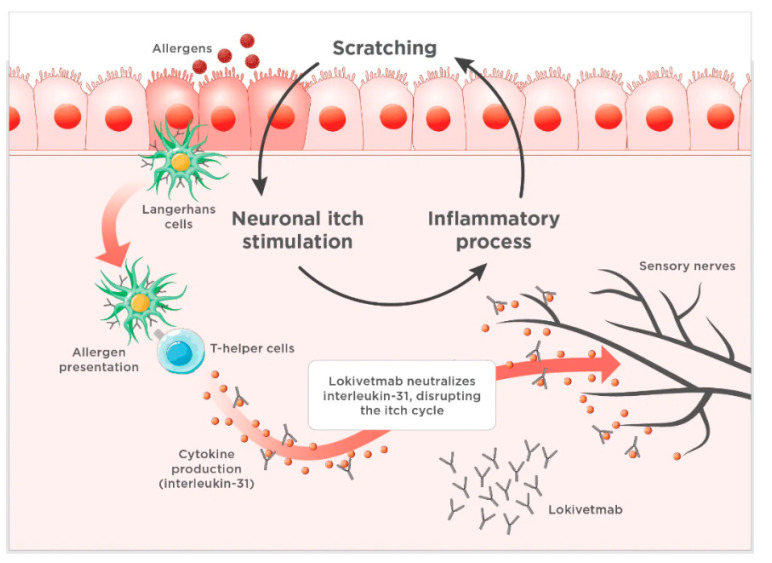
Lokivetmab’s mechanism in disrupting the itch cycle involves targeting canine interleukin-31 (IL-31), which plays a key role in the development of pruritus in dogs suffering from atopic dermatitis. By neutralizing IL-31, lokivetmab effectively reduces itching and helps minimize inflammatory skin lesions (Accessed 27 January 2025. https://todaysveterinarypractice.com/dermatology/advances-in-treatments-for-canine-atopic-dermatitis/).

AD is a long-lasting, complex disorder characterized by inflamed and itchy skin, while allergic dermatitis is a broader term that refers to skin inflammation caused by various allergens, such as food, flea bites, or chemicals. AD impacts 10–15% of dogs overall and has a genetic predisposition [39]. Canine AD requires life-long management and, in most affected dogs, long-term therapy [40]. In the context of canine AD, extensive research into its pathophysiology has identified the primary immunological mediators responsible for pruritus and inflammation: cytokines. These small protein molecules, secreted by T-lymphocytes, play a pivotal role in cell-to-cell interactions and communication [41]. Cytokines contribute to an intricate interconnected network of biochemical interactions, leading to the clinical manifestations associated with AD. Overexpression of canine interleukin-31 (IL-31) has been implicated in the pathophysiology of pruritus in dogs with AD. This includes disruption of the skin barrier function, pruritus, inflammation, and the subsequent development of characteristic clinical lesions. Recent studies have further supported IL-31’s role in the development of pruritus associated with AD [42,43]. Therefore, anti-IL-31 mAb was designed to neutralize free IL-31, primarily produced by T-lymphocytes. Its development stems from the recognition of IL-31’s involvement in canine osteoarthritis [42]. The mechanism of action of anti–IL-31 mAb (lokivetmab) is displayed in Figure 3.

The approval of the first mAb by the USDA for the treatment of AD and allergic dermatitis in dogs—lokivetmab, a caninized, anti–IL-31 mAb—took place in December 2016. Lokivetmab was also the first mAb approved for use in animals in the EU; it was approved by the EMA in 2017 [44] and in Switzerland in 2018. The original label indication of lokivetmab (Cytopoint^®^) was that it was to be used to treat the clinical signs associated with AD in dogs of any age weighing 3 kg or more.

### 5.2. Anti-Nerve Growth Factor mAb for Pain Control in Dogs and Cats—Frunevetmab and Bedinvetmab

Osteoarthritis (OA)-related pain management remains challenging and is a frequent cause of euthanasia in dogs and cats [45]. Conventional therapy for OA pain management in felines and canines consists of weight loss, exercise, acupuncture, massage, and pharmacological measures, including non-steroidal anti-inflammatory drugs (NSAIDs), gabapentin, and tramadol (an opioid). Currently, NSAIDs, particularly cyclooxygenase-2 (COX-2) inhibitors, are commonly used for pain control in dogs and cats. While COX-2 inhibitors are generally safer than NSAIDs with COX-1 inhibitory effects, which are not suitable for dogs or cats, COX-2 inhibitors can still cause adverse effects such as gastrointestinal irritation, kidney dysfunction, and liver toxicity, particularly with long-term use [20,46]. However, pain may not be completely relieved when a NSAID is used alone [47].

**Figure 4:**
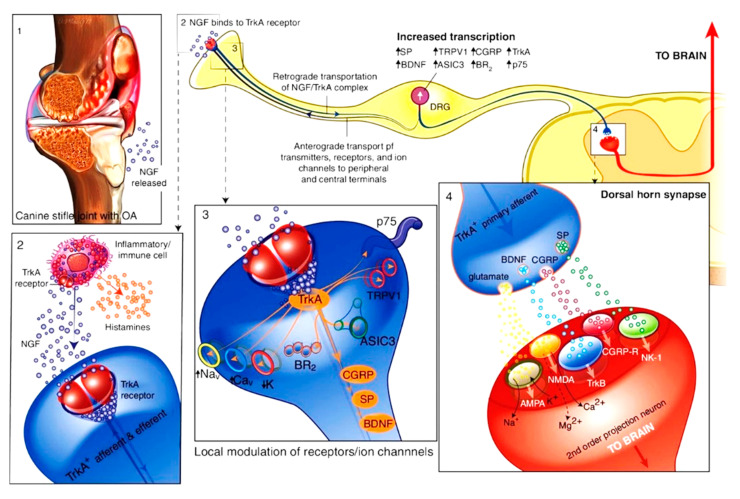
NGF is the key player in pain perception and nervous system plasticity in osteoarthritis. Released by chondrocytes, NGF binds to TrkA on sensory fibers and immune cells, prompting the release of inflammatory mediators/autacoids, e.g., histamine and serotonin. The NGF/TrkA complex is transported to the dorsal root ganglion (DRG), increasing the expression of pain-related receptors and ion channels (e.g., TRPV1, ASIC, Nav), leading to peripheral sensitization. NGF also enhances production of pro-nociceptive neurotransmitters (SP, CGRP, BDNF), which, upon release, stimulate second-order neurons, potentially causing central sensitization. This process amplifies pain signaling from the periphery, e.g., joint to the brain [20]. Abbreviations: 5-HT, serotonin; AMPA, α-amino-3-hydroxy-5-methyl-4-isoxazolepropionic acid; ASIC, acid-sensing ion channel; BDNF, brain-derived neurotrophic factor; BR2, bradykinin receptor 2; Cav, calcium channel; CGRP, calcitonin gene-related peptide; DRG, dorsal root ganglion; K, potassium channel; Nav, sodium channel; NMDA, N-methyl-D-aspartate receptor; NGF, nerve growth factor; SP, substance P; TrkA/B, tropomyosin receptor kinase.

NGF is essential for the growth and maintenance of sensory and sympathetic neurons [48]. NGF and its interaction with tropomyosin receptor kinase A receptor TrkA have been found to play a critical role in nociception and nervous system plasticity, and in pain signaling in pain conditions in mammals (Figure 4) [20,48]. Thus, anti-NGF therapy looks to be both very effective and promising as a novel therapy against chronic pain [20]. The anti-NGF mAbs bind to NGF, preventing the interaction with its receptors, TrkA, and consequently interrupting the NGF/TrkA signaling and decreasing the pain response associated with OA [20]. Therefore, various mAb therapies targeting this pathway have been investigated to develop pharmacotherapies for chronic pain. These mAbs target a canine- and feline-specific NGF [49].

Frunevetmab (NV-02, Solensia^®^), is a felinized immunoglobin G (IgG) mAb that binds to NGF and blocks its pain action in cats. When frunevetmab binds to NGF, it prevents the pain signal from reaching the brain. Frunevetmab was well tolerated in cats; it significantly decreased signs of lameness up to 7 days after induction of inflammation. Bedinvetmab (Librela^®^) is a canine IgG mAb, in which the variable regions from canine B-lymphocyte sequence are joined with canine IgG constant sequences and it is expressed through recombinant DNA techniques in Chinese hamster ovary (CHO) cells. Bedinvetmab is administered SC once a month, targeting NGF. Librela^®^ is the first and only injectable mAb treatment for the control of canine OA pain approved in the US.

### 5.3. MAbs for Treatment of Immunosuppressive Cancers in Dogs-Gilvetmab

Mast cell tumors (MCTs) and melanoma are not only among the most common cancers in dogs, comprising 20% of skin cancer cases [50] and 7% of all malignant tumors [51], respectively, but they are also known to be immunosuppressive [52]. Programmed death receptor-1 (PD-1) is found on activated T-lymphocytes, and its interaction with programmed cell death ligand 1 (PD-L1), present on tumor cells or certain immune cells, hampers T-lymphocyte activities, including the production of interferon-γ. These MCTs and melanoma can evade the immune system by expressing high levels of immune checkpoint molecules such as PD-L1, PD-L2, and PD-1, which inhibit T-lymphocyte function and allow tumor cells to escape immune detection and destruction. The immunosuppressive nature of these tumors provides a rationale for exploring therapies that target these checkpoint molecules, such as mAbs against PD-1, to reactivate the immune response and enhance anti-tumor immunity [53].

**Figure 5:**
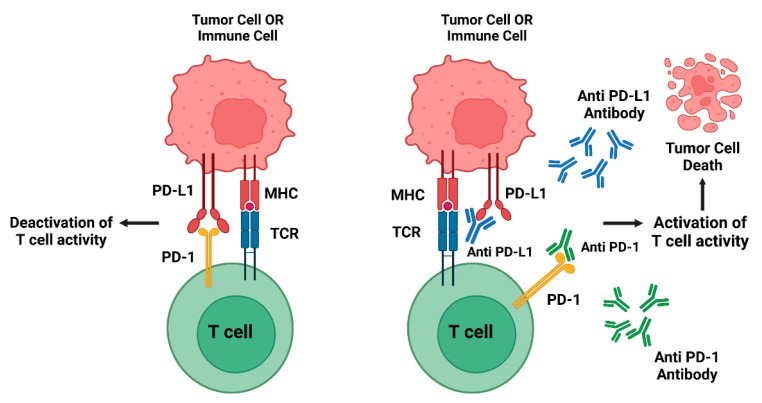
Inhibitors of PD-1 in cancer therapy work by blocking the PD-L1/PD-1 pathway, thereby reactivating the immune system’s anti-tumor response at two critical points: the cognitive phase in lymph nodes and the effector phase within the tumor microenvironment. These inhibitors include anti-PD-1 and anti-PD-L1 antibodies, which effectively disrupt the signaling that allows tumors to evade immune detection [55,57].

In veterinary oncology, research is increasingly focused on developing canine-specific antibodies, with anti-PD-1 and anti-PD-L1 therapies showing significant potential, mirroring the progress made in human cancer treatment (Figure 5) [54,55]. When PD-L1 or PD-L2 binds to its receptor, PD-1, T-lymphocyte is inactivated, and downstream signaling pathways are initiated, leading to immune inhibition [54]. Nemoto et al. (2018) reported that mAb targeted two immune inhibitory checkpoint molecules (i.e., canine PD-1 [cPD-1] and canine PD-L1 [cPD-L1]). These mAbs bind to over-expressed cPD-1 and cPD-L1, respectively, effectively blocking the binding between cPD-1 and cPD-L1. Both mAbs were proven to possess a different blocking ability and a unique functional blockade. These recently manufactured mAbs may confer a new strategy for the treatment of dogs with immunosuppressive cancers [56].

Gilvetmab, developed exclusively for therapeutic use in dogs, is a caninized IgG mAb and checkpoint inhibitor that specifically targets PD-1, reactivating the dog’s immune system to recognize and fight cancer cells. Gilvetmab is used to treat dogs with MCT and melanoma [58]. It is an ideal systemic treatment option labeled for dogs with stages I, II, and III MCT and stages II and III melanoma. Gilvetmab binds cPD-1, interrupting the interaction between PD-L1/L2 and PD-1 [59], thereby enhancing the immune response to fight these cancers [60]. By preventing immune suppression, Gilvetmab allows the dog’s immune system to destroy cancer cells. The initial studies showed that 77.4% of veterinarians reported a quality of life performance score of 0 or 1, indicating excellent improvement of quality of life [58]. A field study demonstrated that gilvetmab is well tolerated in dogs with MCT or melanoma [61]. Adverse reactions are generally transient and include mainly lethargy and inappetence/gastrointestinal disturbance [58]. Gilvetmab received conditional license approval for the treatment of dogs with MCT or melanoma by the USDA CVB (13 October 2023) [62].

### 5.4. Canine Parvovirus MAb

Canine parvovirus (CPV) is a prevalent cause of illness and death in dogs around the globe. This highly contagious and potentially lethal disease impairs the intestinal lining’s ability to absorb nutrients, retain fluids, and combat bacterial infections [63,64]. CPV is one of the most contagious and dangerous viruses that can infect dogs, with a 91% mortality rate if untreated [65]. Although vaccination is strongly recommended and can significantly reduce the risk of CPV infection, there are no effective treatment options for dogs that became infected. It is estimated that there are 330,000 cases of CPV infection available for treatment in the U.S. annually [66].

CPV Monoclonal Antibody (CPMA) is the first USDA-conditionally approved mAb (2 May 2023), one-dose treatment option for CPV infection in dogs 8 weeks of age or older. CMPA consists of a dog constant region and a rat variable region. CPMA binds directly to CPV and neutralizes it before it can enter the cell, preventing viral infiltration into host cells and delivering a high level of defense [67]. When administered to unvaccinated young dogs exposed to CPV, the product increases survival, reduces morbidity, accelerates remission of clinical signs, and shortens hospitalization. The clinical trials demonstrate that the CPMA single IV dose delivers targeted efficacy in treating this deadly disease. However, there is no trade name assigned to this CPMA at the USDA website. The results of this clinical trial were published in a conference abstract and the corresponding published peer-reviewed results are still not available.

### 5.5. Regulatory Approval of Veterinary MAbs in the European Union (EU) and United States (US)

Similar to drugs intended for human use, veterinary drugs must undergo a rigorous registration process in both the EU and US jurisdictions. In the EU, the approval or rejection of veterinary drugs applications is determined by a specialized body known as the Committee for Veterinary Medicinal Products (CVMP), which is analogous to the Committee for Human Medicinal Products (CHMP) responsible for authorizing human drugs [68]. The regulatory framework governing this approval process is outlined in the “REGULATION (EU) 2019/6 OF THE EUROPEAN PARLIAMENT AND OF THE COUNCIL of 11 December 2018 on veterinary medicinal products and repealing Directive 2001/82/EC” [69]. The European Medicines Agency (EMA) issued its first guidance on mAbs for use in animals in 2017 [70]. Similar to the approval process for human products, applications for veterinary drugs derived from biotechnological processes must undergo a centralized procedure.

The regulatory pathway in the US for veterinary mAbs varies depending on their intended use. Biopharmaceutical products can be regulated by the FDA or USDA depending on various factors such as the nature of the molecule, therapeutic target and claim, and mechanism of action. Specifically, the drug’s therapeutic target determines which regulatory agency reviews and approves it. The USDA Center for Veterinary Biologics approves mAbs that target the immune system, while the FDA Center for Veterinary Medicine approves mAbs that have targets other than the immune system. Briefly, if the product is related to immunity, it is under the control of USDA; otherwise, it is under the FDA control. This is why anti-NGF products (frunevetmab and bedinvetmab) are under FDA control, but other products (e.g., lokivetmab) are under USDA control. The USDA and FDA have not yet issued specific guidance for veterinary mAbs. The FDA follows Veterinary International Conference on Harmonization (VICH) principles (https://www.fda.gov/animal-veterinary/guidance-industry/veterinary-international-conference-harmonization-vich-guidance-documents#:~:text=Veterinary%20International%20Conference%20on%20Harmonization%20(VICH)%20Guidance%20Documents%20%7C%20FDA, (accessed on 20 December 2024)), similar to those for human antibodies. Approaching each case flexibly, mAbs approved by the FDA with full license currently include frunevetmab (Solensia^®^) and bedinvetmab (Librela^®^). 

## 6. The Opportunities and Challenges of mAbs in Veterinary Medicine

More than 160 mAbs have been licensed worldwide to treat human diseases such as neoplasms, infectious diseases, chronic inflammatory conditions, and cardiovascular disorders [71]. In contrast, there are only five commercial mAbs available in veterinary medicine. Biotherapeutics and targeted pharmacology represent the future of disease treatment in this field [72]. MAb therapies show promise for expansion into disease areas in veterinary medicine. While mAbs for companion animals offer numerous opportunities, there are still many unmet needs in treating various diseases. The emergence of new technical platforms related to the target selection also facilitates the rapid mAbs development, such as ribosome display methodology, DNA recombinant antibody technique, antibody phage display, and isolation of single B-lymphocytes [73]. Similar to advancements in human medicine, where new modalities such as pAb, bi-specific antibodies, and antibody fragments have demonstrated unique therapeutic efficacy in humans, there is potential to replicate these successes in companion animal therapeutics. For example, antibody fragments are therapeutic proteins used to improve tissue penetration and reduce immunogenicity [74]. Additionally, new expression systems have been employed for the production of mAbs for companion animals, with the goal of reducing production costs.

The development of mAbs for small animal medicine faces several challenges. Unlike human medicine, veterinary medicine has limited knowledge of animal species-specific biology and disease mechanisms. In addition, diseases such as cancers, skin diseases, and renal disorders often have complex, multi-target characteristics that necessitate a diverse array of drugs [75], more effective therapeutics, or combination therapies to achieve optimal outcomes. Additionally, the research and production costs associated with mAb drug development for companion animals can be significant. Investment in research and development for companion animal mAb drugs is relatively limited compared with the human pharmaceutical industry, and the lower profit margins pose an additional hurdle. Clinical studies can also take a long time due to a limited number of animals with specific diseases/conditions and slow recruitment rates. Furthermore, as a developing field, regulatory frameworks for mAb drugs in companion animals are still evolving. Increasing expectations from regulatory agencies may lead to stricter requirements, potentially slowing down drug development and market entry. Providing clearer, science-based regulatory guidance could help facilitate the approval and commercialization of these innovative therapeutics.

## 7. Conclusions

Therapeutic antibodies have achieved great success in both human and animal healthcare. Therapeutic mAbs for companion animals are expected to fill the unmet medical needs in small animal diseases such as inflammatory, neoplastic, autoimmune, and infectious diseases, providing targeted treatment options for companion animals. Unlike pAbs, mAbs offer unparalleled specificity, allowing for tailored treatment strategies that minimize adverse effects on healthy tissues. With advancements in mAb research and manufacturing technologies, the availability and effectiveness of veterinary therapeutic mAbs are expected to gradually increase. In the future, continuous research and development efforts will play an important role in improving the health and well-being of companion animals through the use of mAb therapies.

## Figures and Tables

**Table 1:** Profile of veterinary mAbs approved for use in companion animals (in chronological order).

Drug (Trade Name, Company)	Route of Administration	Target	Format	Storage	Recommended Dose (Frequency)	Labeled for Use Against:
Bedinvetmab (Librela™, Zoetis)	SC	Dog NGF	caninized	2–8 °C	0.5–1.0 mg/kg, once a month	Pain associated with osteoarthritis (OA) in dogs
Canine Parvovirus Monoclonal Antibody (CPMA) (no trade name, Elanco) *	IV	CPV2	Rat–canine chimera	Frozen	0.2 mL/kg	Anti-virus infection (blocking virus entry)
Frunevetmab (Solensia^®^, Zoetis)	SC	Cat NGF	felinized	2–8 °C	1.0 to 2.8 mg/kg every 28 days (once-a-month drug treatment)	Pain associated with OA in cats.
Gilvetmab * (no trade name assigned, Intervet Inc.) Merck Animal Health	IV	Dog PD-1	caninized	2–8 °C	10 mg/kg every 2 weeks for up to 10 treatments	Cancer (mast cell tumors and melanomas)
Lokivetmab (Cytopoint^®^, Zoetis)	SC	Dog IL-31	caninized	2–8 °C	2 mg/kg (US) or 1 mg/kg EU every 4 to 8 weeks as needed.	Allergic and atopic dermatitis in dogs.

* Conditional license from USDA.

## Data Availability

No new data were created or analyzed in this study. Data sharing is not applicable to this article.

## Abbreviations

The following abbreviations are used in this manuscript:

AD	Atopic dermatitis
ADA	Anti-drug antibody
ADCC	Antibody-dependent cellular cytotoxicity
CD20	also known as cluster of differentiation 20, is a cell-surface protein expressed primarily on B-lymphocytes.
CDC	Complement-dependent cytotoxicity
CDR	Complementarity-determining regions
CPMA	Canine parvovirus monoclonal antibody
FcRn	Neonatal Fc receptor
FcγR	Fc-gamma receptors
FDA	Food and Drug Administration
mAbs	Monoclonal antibodies
MCTs	Mast cell tumors
NAbs	Neutralizing antibodies
NSAIDs	Non-steroidal anti-inflammatory drugs
NGF	Nerve growth factor
OA	Osteoarthritis
pAbs	Polyclonal antibodies
TrkA	Tropomyosin receptor kinase A receptor
USDA	United States Department of Agriculture
